# Supplementary material for: Adaptation and Validation of the Program Sustainability Assessment Tool (PSAT) for Use in the Elementary School Setting
Source: Int J Environ Res Public Health. 2021 Oct 29;18(21):11414. doi: 10.3390/ijerph182111414 (PMC8582873; doi:10.3390/ijerph182111414)
Supplement: Supplementary file 1 [file ijerph-18-11414-s001.zip › ijerph-1410714-SI.pdf]

Table S1. Comparison of the original PSAT items to the adapted items for use in the elementary school setting

Original PSAT item	Amended item	Rationale for amendment
**Domain: Strategic planning**		
The program plans for future resource needs		This item is not relevant for schools as educational departments, school boards or executives are usually responsible for determining long term resource needs.
The program has a long-term financial plan.		This item is not relevant for schools as educational departments, school boards or executives are usually responsible for determining long term financial resources.
The program has a sustainability plan.	My school has a sustainability plan (e.g. to continue the scheduling of the recommended minutes of physical activity long-term).	Wording change to make relevant to the school setting and emphasise the specific intervention being sustained (i.e. the Department of Education PA policy).
The program's goals are understood by all stakeholders.	My school's goals to maintain the scheduling of PA are understood by all stakeholders (e.g. teachers, school champions, principals).	Wording change to make relevant to the school setting and emphasise the specific intervention being sustained (i.e. the Department of Education PA policy).
The program clearly outlines roles and responsibilities for all stakeholders.	My school clearly outlines roles and responsibilities to schedule PA for all stakeholders (e.g. teachers, school champions, principals).	Wording change to make relevant to the school setting and emphasise the specific intervention being sustained (i.e. the Department of Education PA policy).
**Domain: Environmental support**		
Champions exist who strongly support the program.	There are champions within the school advocating for the scheduling of PA *(a champion is someone who supports and advocates the policy, this may be your school executive or a teacher within the school).*	Wording change to make relevant to the school setting and emphasise the specific intervention being sustained (i.e. the Department of Education PA policy).<br><br>An example of a school champion was also included to reduce confusion by respondents.
The program has strong champions with the ability to garner resources.	There are champions within the school with the ability to get resources for the scheduling of PA.	Wording change to make relevant to the school setting and emphasise the specific intervention being sustained (i.e. the Department of Education PA policy).

The program has leadership support from within the larger organization.	My school has support from within the broader organisation i.e. DoE/ CSO for the scheduling of PA.	Wording change to make relevant to the school setting and emphasise the specific intervention being sustained (i.e. the Department of Education PA policy).
The program has leadership support from outside of the organization.	My school has support from outside our education department/office to help the scheduling of PA.	Wording change to make relevant to the school setting and emphasise the specific intervention being sustained (i.e. the Department of Education PA policy).
The program has strong public support.	The scheduling of PA for students at my school has strong public and community support.	Wording change to make relevant to the school setting and emphasise the specific intervention being sustained (i.e. the Department of Education PA policy).
**Domain: Program adaptation**		
The program periodically reviews the evidence base.		This item is not relevant for schools as educational departments and school boards are usually responsible for determining the programs delivered to schools.
The program adapts strategies as needed.	My school adapts or changes the scheduling of physical activity each week as needed (e.g. if PE equipment is damaged and cannot be used, heat wave etc.).	Wording change to make relevant to the school setting and emphasise the specific intervention being sustained (i.e. the Department of Education PA policy).
The program adapts to new science.		This item is not relevant for schools as educational departments and school boards are usually responsible for determining the programs delivered to schools.
The program proactively adapts to changes in the environment.	My school has a process to proactively adapt the scheduling of PA to meet changes in needs of the school community (e.g. to include other school programs).	Wording change to make relevant to the school setting and emphasise the specific intervention being sustained (i.e. the Department of Education PA policy).
The program makes decisions about which components are ineffective and should not continue.	My school makes decisions about which physical activity components are ineffective and should not continue when scheduling PA (e.g. energizers, GoNoodle, running etc.)	Wording change to make relevant to the school setting and emphasise the specific intervention being sustained (i.e. the Department of Education PA policy).
**Domain: Organisational capacity**		

The program is well integrated into the operations of the organization.	The scheduling of PA is well integrated into the operations of our school.	Wording change to make relevant to the school setting and emphasise the specific intervention being sustained (i.e. the Department of Education PA policy).<br><br>*This item was later removed from the adapted PSAT due to high correlation and potential redundancy with item "School systems (e.g. space, time allocation) are in place to support the scheduling of PA."*
Organizational systems are in place to support the various program needs.	School systems (e.g. space, time allocation) are in place to support the scheduling of PA.	Wording change to make relevant to the school setting and emphasise the specific intervention being sustained (i.e. the Department of Education PA policy).
Leadership effectively articulates the vision of the program to external partners.		This item is not relevant for schools as educational departments and school boards are usually responsible for determining the programs delivered to schools.
Leadership efficiently manages staff and other resources	School executives manage staff and other resources effectively to ensure that the scheduling of PA is met.	Wording change to make relevant to the school setting and emphasise the specific intervention being sustained (i.e. the Department of Education PA policy).
The program has adequate staff to complete the program's goals.	My school has enough trained school champions to support the scheduling of PA.	The successful delivery of most school health programs is not reliant on the number of teaching staff, but whether there are an adequate number of trained school champions to support teachers in their delivery.
	There are adequate resources and infrastructure within the school to schedule PA.	Identified as an important factor to ensuring the ongoing delivery of health programs in schools.
	School champions and teachers at my school have enough supervision and support to implement the scheduling PA.	Identified as an important factor to ensuring the ongoing delivery of health programs in schools.
	The level of school champion / teacher turnover is	Identified as an important factor to ensuring the

	manageable to sustain the scheduling of PA.	ongoing delivery of health programs in schools.<br><br>*This item was later removed from the adapted PSAT based on modification indices indicating correlation with item "School champions and teachers at my school have enough supervision and support to implement the scheduling PA."*
	My school has a system for training new school champions / teachers to schedule PA.	Identified as an important factor to ensuring the ongoing delivery of health programs in schools.<br><br>*This item was later removed from the adapted PSAT based on modification indices indicating cross-loading with the Program Evaluation domain.*
**Domain: Communications**		
The program has communication strategies to secure and maintain public support.	My school has communication strategies in place to secure and maintain our school communities' support for scheduling PA.	Wording change to make relevant to the school setting and emphasise the specific intervention being sustained (i.e. the Department of Education PA policy).
Program staff communicate the need for the program to the public.	Staff members at my school communicate the need for scheduling PA to the community (E.g. parents)	Wording change to make relevant to the school setting and emphasise the specific intervention being sustained (i.e. the Department of Education PA policy).
The program is marketed in a way that generates interest.		This item is not relevant for schools as educational departments and school boards are usually responsible for determining the programs delivered to schools.
The program increases community awareness of the issue.	My schools' scheduling of PA increases community awareness of the need for PA in children	Wording change to make relevant to the school setting and emphasise the specific intervention being sustained (i.e. the Department of Education PA policy).
The program demonstrates its value to the public.		This item is not relevant for schools as educational departments and school boards are usually responsible for determining

		the programs delivered to schools.
**Domain: Program evaluation**		
The program has the capacity for quality program evaluation.	My school has a system in place to actively evaluate the scheduling of PA (e.g. Improvements in children's PA, student on-task behaviour etc.)	Wording change to make relevant to the school setting and emphasise the specific intervention being sustained (i.e. the Department of Education PA policy).
The program reports short term and intermediate outcomes.	My school reports the outcomes of scheduling the recommended minutes of PA (e.g. Improvement in student physical activity levels)	Wording change to make relevant to the school setting and emphasise the specific intervention being sustained (i.e. the Department of Education PA policy).
Evaluation results inform program planning and implementation.	Evaluation results inform the planning and implementation of the scheduling of PA.	Wording change to make relevant to the school setting and emphasise the specific intervention being sustained (i.e. the Department of Education PA policy).
Program evaluation results are used to demonstrate successes to funders and other key stakeholders.	Evaluation results of the scheduling of PA are used to demonstrate success to funders and other key stakeholders (e.g. P&C, wider school community, etc.)	Wording change to make relevant to the school setting and emphasise the specific intervention being sustained (i.e. the Department of Education PA policy).<br><br>*This item was later removed from the adapted PSAT due to high correlation and potential redundancy with items "My school has a system in place to actively evaluate the scheduling of PA (e.g. Improvements in children's PA, student on-task behaviour etc.)" and "My school reports the outcomes of scheduling the recommended minutes of PA (e.g. Improvement in student physical activity levels)" and "Evaluation results inform the planning and implementation of the scheduling of PA.".*
The program provides strong evidence to the public that the program works.		This item is not relevant for schools as educational departments and school boards are usually responsible for determining

		the programs delivered to schools.
**Domain: Funding stability**		
The program exists in a supportive state economic climate.		This item is not relevant for schools as educational departments, school boards or executives are usually responsible for examining the economic climate.
The program implements policies to help ensure sustained funding.	The school takes action to ensure there are ongoing funds to support the scheduling of PA. (e.g. included in annual school budget, funding from P&C)	Wording change to make relevant to the school setting and emphasise the specific intervention being sustained (i.e. the Department of Education PA policy).
The program is funded through a variety of sources.	My school can access a variety of funding sources to help schedule PA.	Wording change to make relevant to the school setting and emphasise the specific intervention being sustained (i.e. the Department of Education PA policy).
The program has a combination of stable and flexible funding.		This item is not relevant for schools as educational departments, school boards or executives are usually responsible for determining long term financial resources.
The program has sustained funding.		This item is not relevant for schools as educational departments, school boards or executives are usually responsible for determining long term financial resources.
	My school has a process in place to allow staff to attend professional development on scheduling PA (i.e. funding for ongoing professional development)	Identified as an important factor to ensuring the ongoing delivery of health programs in schools.
	My school provides time at work for staff to plan their schedule for meeting the recommended minutes of PA.	Identified as an important factor to ensuring the ongoing delivery of health programs in schools.
**Domain: Partnerships**		
Diverse community organizations are invested in the success of the program.		This item is not relevant for schools as programs are often determined by educational departments or boards, and thus the establishment of partnership with stakeholders was considered outside the scope of individual schools.

The program communicates with community leaders.		This item is not relevant for schools as programs are often determined by educational departments or boards, and thus the establishment of partnership with stakeholders was considered outside the scope of individual schools.
Community leaders are involved with the program.		This item is not relevant for schools as programs are often determined by educational departments or boards, and thus the establishment of partnership with stakeholders was considered outside the scope of individual schools.
Community members are passionately committed to the program.		This item is not relevant for schools as programs are often determined by educational departments or boards, and thus the establishment of partnership with stakeholders was considered outside the scope of individual schools.
The community is engaged in the development of program goals.		This item is not relevant for schools as programs are often determined by educational departments or boards, and thus the establishment of partnership with stakeholders was considered outside the scope of individual schools.
